# SCN4B inhibits the progression of lung adenocarcinoma and is associated with better prognosis

**DOI:** 10.1111/crj.13709

**Published:** 2023-10-12

**Authors:** Minting Ma, Bin Guo, Hongwei Lu, Lei Hong

**Affiliations:** ^1^ Department of Oncology The Fourth Hospital of Hebei Medical University Shijiazhuang Hebei Province China; ^2^ Department of Thoracic Surgery The Fourth Hospital of Hebei Medical University Shijiazhuang Hebei Province China; ^3^ Department of Ophthalmology The Fourth Hospital of Hebei Medical University Shijiazhuang Hebei Province China

**Keywords:** ERG, lung adenocarcinoma, prognosis, SCN4B, TAL1

## Abstract

**Introduction:**

Lung adenocarcinoma (LUAD) is the major type of non‐small cell lung cancer with low a survival rate caused by metastasis. SCN4B encoding voltage‐gated sodium channel β subunit is regarded as a metastasis‐suppressor gene. We aim to explore how SCN4B influences the progression and prognosis of LUAD.

**Methods:**

The gene expression profiles of 585 LUAD samples in TCGA and GSE31210, GSE116959, and GSE72094 datasets from the GEO database were downloaded for analysis. Differentially expressed genes were obtained through the “limma” package. The “clusterProfiler” package was used to conduct GSEA. Survival analysis was conducted via “survival” and “survminer” packages. Transcription factors regulating SCN4B expression were screened by correlation analysis and further predicted by FIMO. Infiltration of immune cells was analyzed by CIBERSORT. ESTIMATE algorithm was used to evaluate the immune‐related scores.

**Results:**

SCN4B expressed higher in normal samples than in LUAD samples and higher in female samples than male samples. One hundred and twenty‐six pathways were significantly enriched between high and low SCN4B expression groups. Six transcription factors' expressions were positively related to SCN4B expression, and ChIP‐seq data from “Cistrome” verified that TAL1 and ERG might bind to the upstream sequence of SCN4B. SCN4B expression was significantly correlated with activated memory CD4 T cells, resting mast cells, and monocytes. TMB status, three scores based on ESTIMATE algorithm, and expression of three immune checkpoints showed significant differences between SCN4B high‐ and low‐expression groups. SCN4B could be considered as an independent prognostic signature of LUAD patients that higher expression represents a better prognosis.

**Conclusion:**

SCN4B expresses higher in normal samples, and SCN4B is able to be an independent prognostic signature for LUAD patients. TAL1 and ERG may regulate the expression of SCN4B by binding its upstream sequences. Our research is valuable in improving the effectiveness of treatment in LUAD.

## Graphical abstract

SCN4B encoding voltage‐gated sodium channel β subunit is regarded as metastasis‐suppressor gene, and it expresses higher in normal samples than lung adenocarcinoma (LUAD) samples. SCN4B is an independent prognostic signature for LUAD patients, and its higher expression in LUAD samples represents better prognosis. Transcription factors TAL1 and ERG may regulate the expression of SCN4B by binding its upstream sequences.

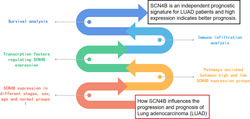

## 1 INTRODUCTION

Lung cancer is one of the leading causes of cancer death with 1.2 million new cases arising annually.^1^ Almost 80% of lung cancers are non‐small cell lung cancers (NSCLCs), and 40% of them are lung adenocarcinoma (LUAD), which is mainly caused by smoking.^2^ The reason for low survival rate despite prevention and treatment is mostly distant metastasis. LUAD strongly trends to metastasize to the brain.^3^ LUAD shows high rates of somatic mutation and genomic rearrangement,^4^ which affect key pathways in LUAD.^5^ Tumors of some patients harbor somatically activated oncogenes such as mutant EGFR1, and molecularly targeted therapies have improved treatment for them.^6^ Alterations not only accumulate in oncogenes but also in suppressor genes in cancer lines during evolution.^7^ It is a long way to understand more about suppressor genes in LUAD for more effective treatment, although there has been a lot of research on LUAD suppressor genes, such as PRDM16,^8^ MIR99AHG,^9^ and TNNC1.^10^

Voltage‐gated sodium channels (VGSCs) containing α and β subunits in mammals work for the initiation and propagation of action potentials in excitable cells.^11^ Besides electrical excitability, β subunits regulate adhesion, migration, pathfinding, and transcription by mediating multiple signaling pathways on different timescales.^12^ There are five β subunits in mammals, namely, β1, β1B, β2, β3, and β4, encoded by four genes: SCN1B–SCN4B.^13^ Reduced β4 protein (encoded by SCN4B) levels in breast cancer biopsies correlate with high‐grade primary and metastatic tumors. In contrast, SCN4B overexpression can reduce cancer cell invasiveness and tumor progression.^14^ When SCN4B is inhibited, colorectal cancer cell proliferation and metastasis are promoted.^15^ Preserved SCN4B expression is an independent indicator of favorable recurrence‐free survival in classical papillary thyroid cancer.^16^ These in vivo and in vitro experiments indicate that SCN4B represents a metastasis‐suppressor gene. In NSCLC, VGSCs might be an integral component of the metastatic process by regulating intracellular sodium homeostasis.^17^, ^18^

Metastasis is one of the reasons for the low survival rate in LUAD, and SCN4B is proven to be a metastasis‐suppressor gene in many cancers, including lung cancer. Hence, we aim to explore how SCN4B influence the progression of LUAD and the prognosis, further aid to improve treatment.

## 2 MATERIALS AND METHODS

### 2.1 Datasets collection

We downloaded gene expression microarray data of 585 LUAD in The Cancer Genome Atlas (TCGA; https://tcga-data.nci.nih.gov/tcga/) database, including 60 normal samples and 525 tumor samples. There were 501 samples with complete survival information for clinical information (Table 1). At the same time, the maf file of LUAD and CNV data for 516 LUAD samples was downloaded for subsequent analysis.

**TABLE 1:** Clinicopathological characteristics of LUAD patients from TCGA‐LUAD database.

Characteristics		Patients (*N* = 501)	
		No.	%
Gender	Female	271	54.09%
	Male	230	45.91%
Age	≤66 (median)	259	51.70%
	>66 (median)	242	48.30%
Grade	I	269	53.69%
	II	119	23.75%
	III	80	15.97%
	IV	25	4.99%
	Unknown	8	1.60%
Survival time	Long (>5 years)	251	50.10%
	Short (<5 years)	52	10.38%
OS status	Dead	182	36.33%
	Alive	319	63.67%
Radiation	Yes	416	83.03%
	No	71	14.17%
	Unknown	14	2.79%
Tobacco	Yes	58	11.58%
	No	361	72.06%
	Unknown	82	16.37%

In addition, we also downloaded the datasets GSE31210 (Affymetrix Human Genome U133 Plus 2.0 Array), GSE116959 (Agilent‐039494 SurePrint G3 Human GE v2 8x60K Microarray 039381), and GSE72094 (Rosetta/Merck Human RSTA Custom Affymetrix 2.0 microarray) from the Gene Expression Omnibus (GEO database; https://www.ncbi.nlm.nih.gov/geo/). There were 226 tumor samples and 20 normal samples in the GSE31210 dataset, 210 of which contained valid survival information. The GSE116959 dataset consisted of 57 tumor samples and 11 normal samples, and 398 tumor samples with complete and valid survival information were included in the GSE72094 dataset.

### 2.2 Identification of differentially expressed genes (DEGs)

R package “limma” (http://master.bioconductor.org/packages/limma/) was used to identify differentially expressed genes (DEGs) between tumor and normal samples. The criteria of |log_2_(fold change [FC])| > 1.0 and adjusted *p*‐value <0.05 were used to filtrate the DEGs. The immunohistochemistry (IHC) results from the Human Protein Atlas (HPA; https://www.proteinatlas.org/) database were downloaded for validation.

### 2.3 Gene set enrichment analysis (GSEA)

The samples in the TCGA dataset were divided into two groups by the median expression level of the target gene. The group with expression levels higher than the median was classified as the high‐expression group (HEG), and the other was the low‐expression group (LEG). The DEGs between HEG and LEG were calculated by the “limma” package. Then R package “clusterProfiler”^19^ (http://master.bioconductor.org/packages/clusterProfiler/) was used to conduct gene set enrichment analysis (GSEA), and the standard of |NES| > 1 and *p*‐value <0.05 was used to screen significantly enriched pathways.

### 2.4 Survival analysis

Survival analyses were performed using the Kaplan–Meier method and the log‐rank test by the survival package (https://CRAN.R-project.org/package=survival) and survminer package to estimate the overall survival (OS) rate of different groups. A multivariate Cox regression model was used to analyze whether the target gene could predict the survival of LUAD patients independently of other factors.

### 2.5 Immune cell infiltration analysis

CIBERSORT (Cell‐type Identification By Estimating Relative Subsets Of RNA Transcripts)^20^ was used to calculate the relative proportions of 22 immune cells from leukocyte gene signature matrix (LM22) in each EwS sample. LM22 contains 547 genes that distinguish 22 human hematopoietic cell phenotypes, including naive and memory B cells, seven T‐cell types, NK cells, plasma cells, and myeloid subsets.^20^ The immune score of the samples was calculated using the “estimate” function package (https://R-Forge.R-project.org/projects/estimate/).

### 2.6 Screening of transcription factors related to SCN4B gene expression

We performed differential expression analysis in LUAD and adjacent samples in the TCGA‐LUAD dataset, and the transcription factors with significant differential expressions were screened according to |log_2_FC| > 1 and *p*‐value <0.05. Then the correlation between transcription factors and SCN4B mRNA was calculated using spearman correlation, and transcription factors significantly related to SCN4B were screened according to *p* < 0.05 and Rho > 0.68.

### 2.7 Prediction of transcription factor binding sites

We download the sequence of 1000 bp upstream of the start site of the SCN4B gene from UCSC (http://genome.ucsc.edu/) and the motif files corresponding to transcription factors from the JASPER database (https://jaspar.genereg.net/). Then we used the online tool FIMO (https://meme-suite.org/meme/tools/fimo) to predict whether there was a transcription factor binding motif in the upstream region of the SCN4B promoter.

### 2.8 Statistical analysis

The Wilcoxon rank sum test was conducted to compare the expression differences of SCN4B in LUAD and normal samples as well as other clinicopathological features. Multivariate Cox regression proportional hazards model determined the independent prognostic indicators for LUAD. When the *p*‐value is less than 0.05, the difference is considered to be statistically significant. We used R language (version 4.1.0) to perform all statistical analyses.

## 3 RESULTS

### 3.1 SNC4B significantly downregulated in LUAD samples

**FIGURE 1:**
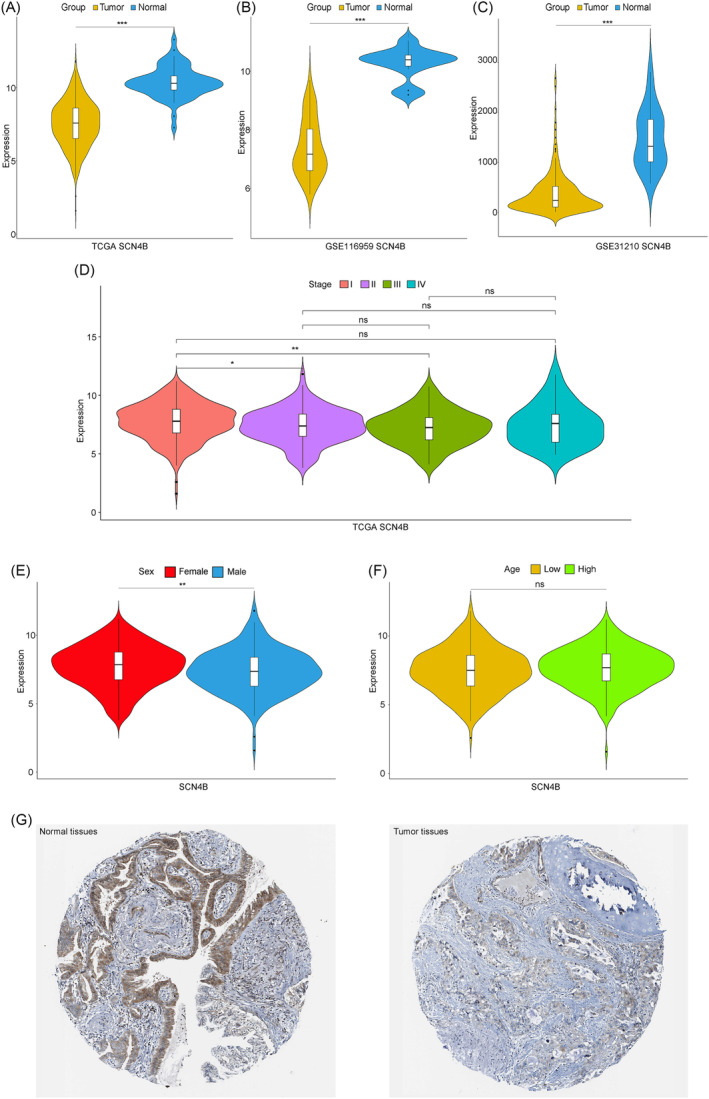
Expression of SCN4B in LUAD and normal samples. (A–C) Differential expression of SCN4B in TCGA, GSE116959, and GSE31210 datasets. (D) Expression of SCN4B in different pathological stages (I, II, III, IV) in clinical information. (E,F) Differential expression of SCN4B in age and gender. (G) Slices of SCN4B expression normal and pathological tissues in the HPA database. The significance levels are: ns represents *p* > 0.05, * represents *p* ≤ 0.05, ** represents *p* ≤ 0.01, *** represents *p* ≤ 0.001, **** represents *p* ≤ 0.0001.

Through differential expression analysis between tumor and normal samples in TCGA‐LUAD, SCN4B was found to be significantly lower expressed in cancer samples (Figure 1A). This trend was further validated by other two lung cancer datasets (GSE116959 and GSE31210) (Figure 1B,C). The expression of SCN4B in tumor samples with different pathological stages (I, II, III, IV) was analyzed, and the results showed the expression level became much lower as the stage passed by from stage I to stage III (Figure 1D). Considering gender and age, SCN4B expression in the female group was significantly higher than those in the male group (Figure 1E), while there was no significant difference in relatively young and old groups (median = 66 years old) (Figure 1F). The IHC results in HPA database showed that SCN4B expressed higher in normal tissues than in tumor tissues in tissue level (Figure 1G). These results showed that the expression of SCN4B in LUAD samples was significantly downregulated.

### 3.2 Pathways changes between high and low SCN4B expression groups

**FIGURE 2:**
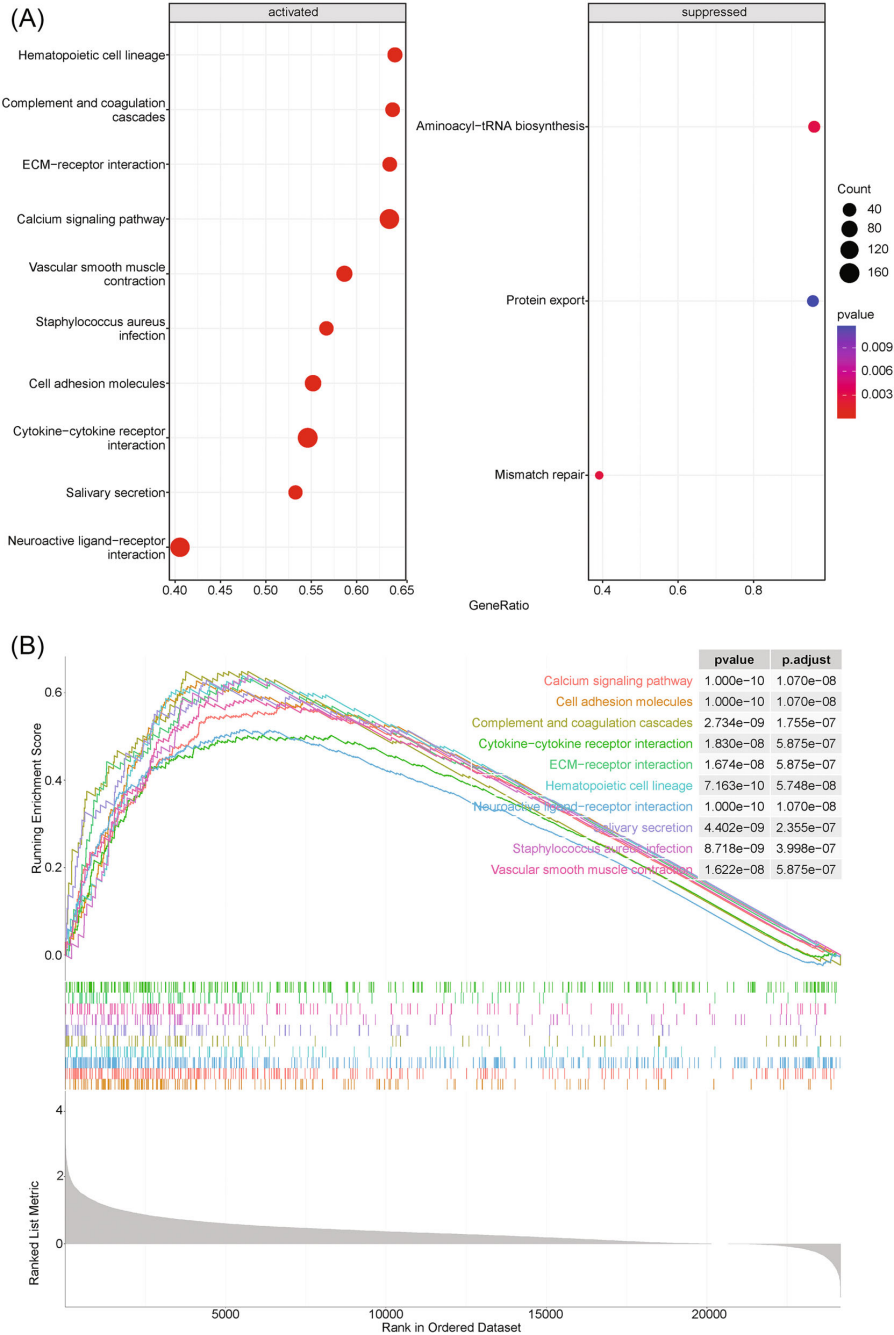
GSEA of LUAD samples of HEG and LEG. (A) The top 10 activated and inhibited pathways. (B) Ten pathways with the smallest *p*‐value.

According to the median expression level of SCN4B, LUAD samples in the TCGA‐LUAD dataset were divided into two groups (HEG and LEG), and GSEA was performed. The results showed that 126 KEGG pathways were significantly enriched in the gene SCN4B HEG compared to LEG (Table S1). The top 10 activated pathways were shown in Figure 2A, including cell adhesion molecules, calcium signaling pathway, neuroactive ligand–receptor interaction, and so on. Three pathways (mismatch repair, aminoacyl‐tRNA biosynthesis, and protein export) were significantly inhibited in the SCN4B LEG group. Ten pathways with highest significance were shown in Figure 2B.

### 3.3 Transcription factors ERG and TAL1 probably binded to SCN4B to regulate its expression

**FIGURE 3:**
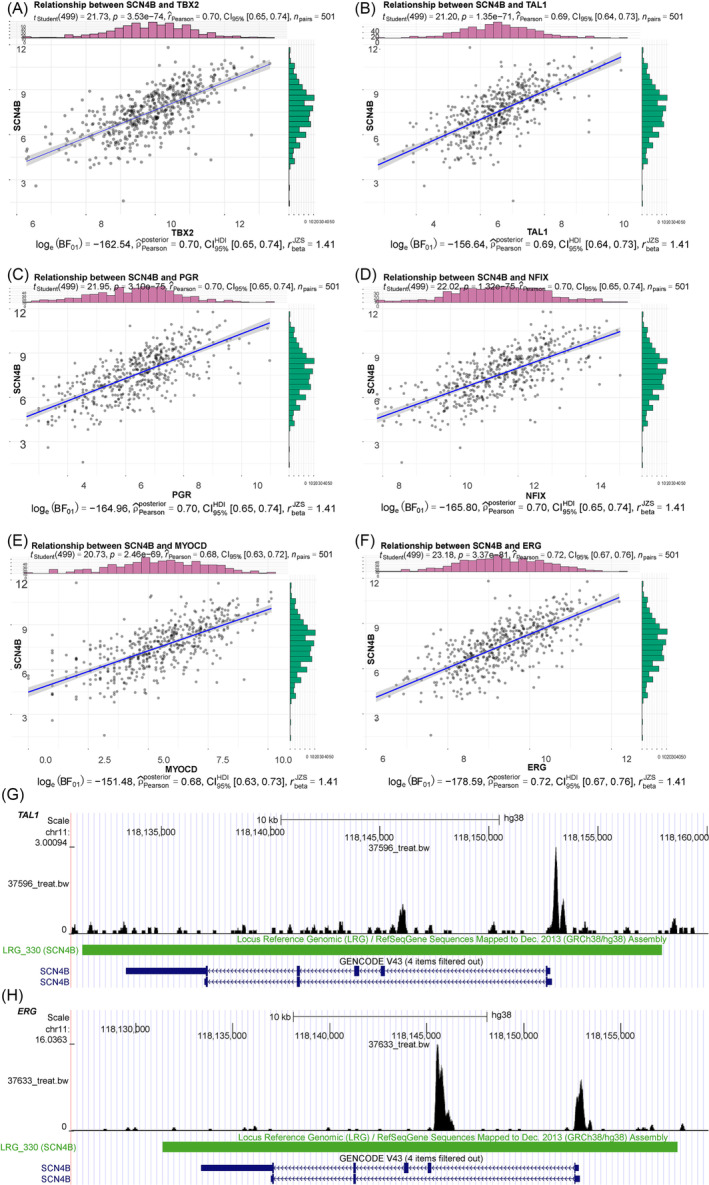
Transcription factors ERG and TAL1 combine with SCN4B to regulate its expression. (A–F) Analysis results of the correlation between the six most significantly correlated transcription factors (TBX2, TAL1, PGR, NFIX, MYOCD, ERG) and SCN4B mRNA. (G,H) ChIP‐seq database results.

We selected a total of 212 transcription factors that were differentially expressed in LUAD samples (Table S2) and calculated the correlation between the 212 transcription factors and SCN4B in the TCGA‐LUAD dataset. According to the standard of *p* < 0.05 and |correlation| > 0.68, we finally found that six transcription factors were significantly positively correlated with the expression of SCN4B (Figure 3A–F). Then we searched for transcription factor binding sequences in the upstream 1000 bp region of the SCN4B promoter. According to *p*‐value < 10^−4^, we found that there might be a binding site of transcription factor T‐cell acute lymphocytic leukemia 1 (TAL1, MA0091.1.meme) at about 835 bp upstream of the SCN4B promoter, a binding site of transcription factor nuclear factor 1 X‐type (NFIX, MA1528.1.meme) at about 574 bp upstream of the SCN4B promoter, and a binding site of transcription factor erythroblast transformation‐specific transcription factor ERG (ERG, MA0474.3.meme) at about 170 bp upstream of the SCN4B promoter, suggesting that the transcription factors TAL1, NFIX, and ERG could regulate the expression of the SCN4B by binding to its upstream sequences (Table S3). Furthermore, by searching the ChIP‐seq public database Cistrome (http://cistrome.org/db/#/), we found that there was an obvious binding peak of TAL1 on SCN4B in Leukemia Cell dataset GSM1122311^21^ (score = 2.594) (Figure 3G). In the Breast dataset GSM726982^22^ (score = 2.391), the ERG ChIP‐seq results showed an obvious binding peak on SCN4B (Figure 3H). The results verified the reliability of TAL1 and ERG regulating the expression of SCN4B by binding with it.

### 3.4 Correlation between SCN4B and immune infiltration in LUAD

**FIGURE 4:**
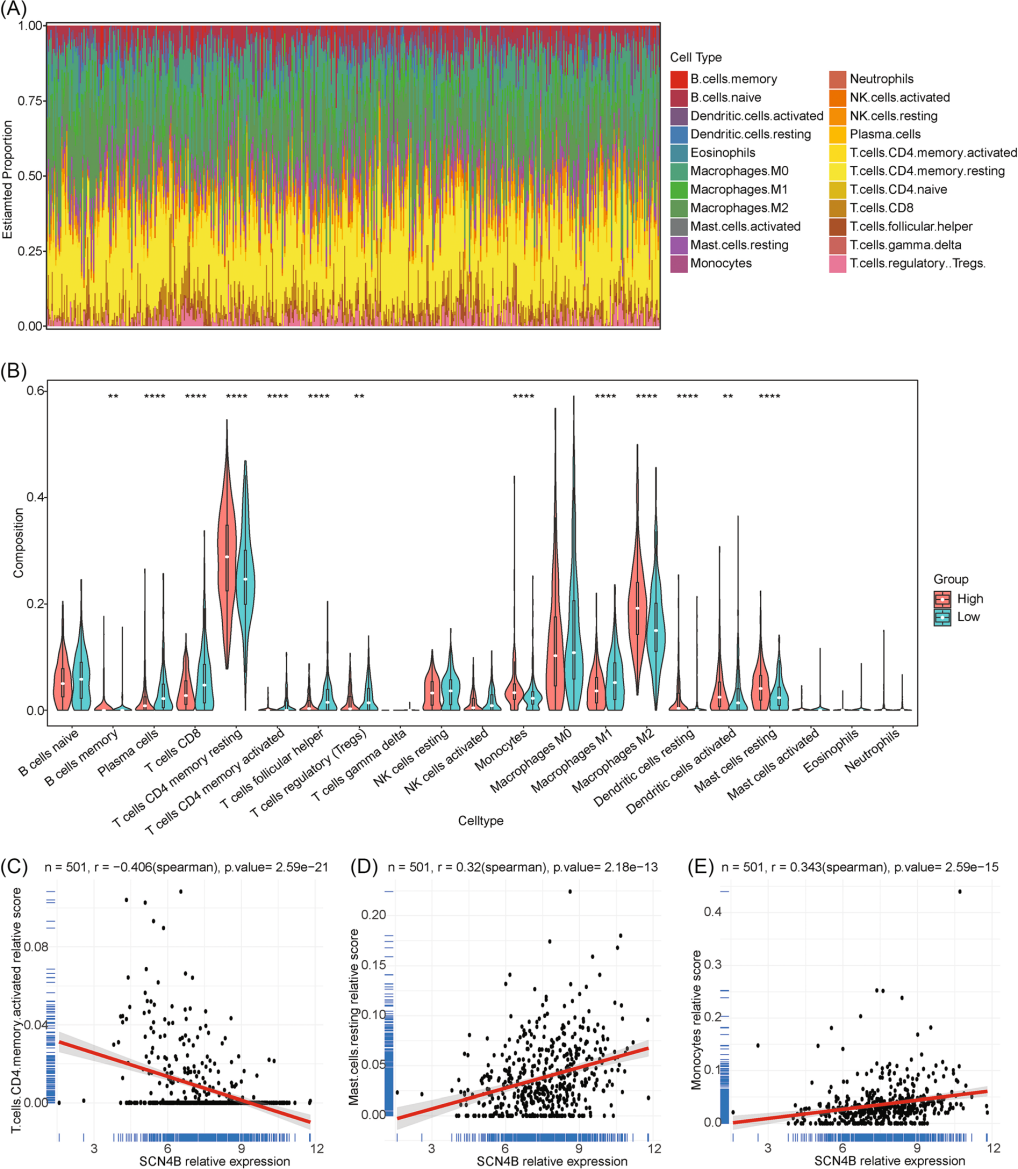
Infiltration of immune cells. (A) Relative content of 22 types of immune infiltrating cells. (B) Difference in immune cell infiltration between 21 types of immune‐infiltrating cells (excluding all zero immune‐infiltrating cells) in samples of HEG and LEG. ** represents *p* ≤ 0.01, *** represents *p* ≤ 0.001, **** represents *p* ≤ 0.0001. (C–E) Correlation of SCN4B's expression and three significantly different immune cells, activated memory CD4 T cells, resting mast cells, and monocytes.

The infiltration of 22 types of immune cells in the TCGA‐LUAD samples was calculated by CIBERSORT (Figure 4A). The difference in infiltration ratios of 21 types of immune cells between HEG and LEG samples was analyzed (immune cells with infiltration ratio of 0 was deleted). The infiltration ratios of 13 types of immune cells were significantly different between samples in HEG and LEG, including memory B cells, plasma cells, CD8 T cells, resting memory CD4 T cells, activated memory CD4 T cells, focal helper T cells, regulatory T cells (Tregs), monocytes, macrophages M1, macrophages M2, resting dendritic cells, activated dendritic cells, and resting mast cells (RMCs) (Figure 4B). Further analysis of the Pearson correlation between SCN4B and the mentioned 13 types of immune cells showed that the expression of SCN4B was significantly correlated with activated memory CD4 T cells, RMCs, and monocytes (|cor| > 0.3 and *p* < 0.05) (Figure 4C–4E).

### 3.5 SCN4B was an independent prognosis factor for LUAD patients

**FIGURE 5:**
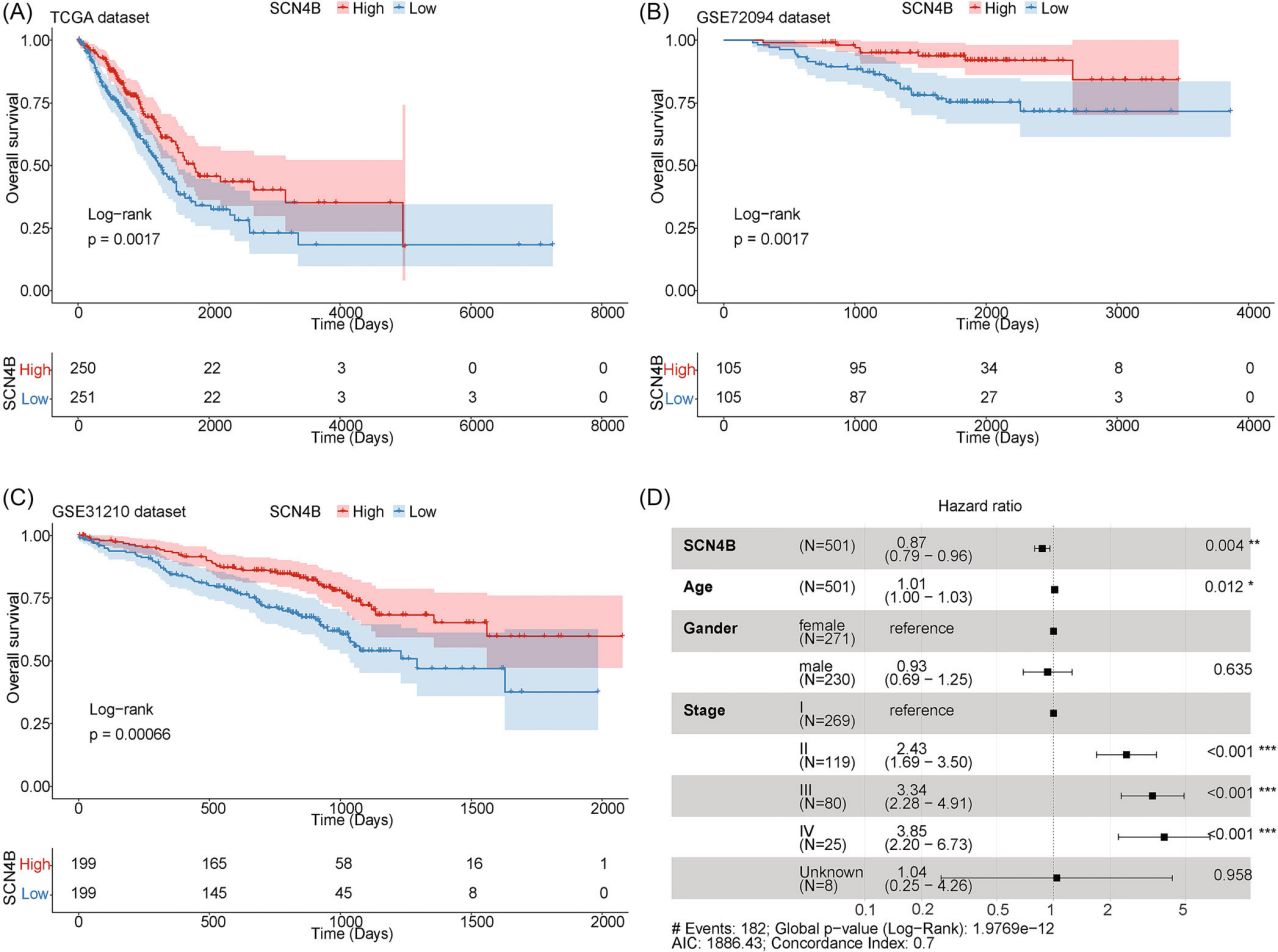
Relationship between expression of SCN4B and prognosis and clinical traits. (A–C) KM survival curves of HEG and LEG in TCGA, GSE72094, and GSE31210 datasets. (D) Multivariate Cox regression analysis. Samples with a hazard ratio greater than 1 have a higher risk of death, and samples with a hazard ratio less than 1 have a lower risk of death compared with reference samples.

Survival analysis was performed on the LUAD patients in the TCGA‐LUAD, and the results showed that the prognosis of patients with low expression of SCN4B was relatively poor (Figure 5A). Survival information from lung cancer datasets GSE72094 and GSE31210 confirmed this result once again (Figure 5B,C). To determine whether SCN4B expression was an independent prognostic factor, clinical information (age, gender, stages) and SCN4B expression value were included for multivariate Cox regression analysis. The result showed that SCN4B was an independent predictor of the outcome of LUAD patients (HR: 0.87 [0.79–0.96] and *p*‐value < 0.01) (Figure 5D).

### 3.6 Differences of mutation landscape between high and low SCN4B expression groups

**FIGURE 6:**
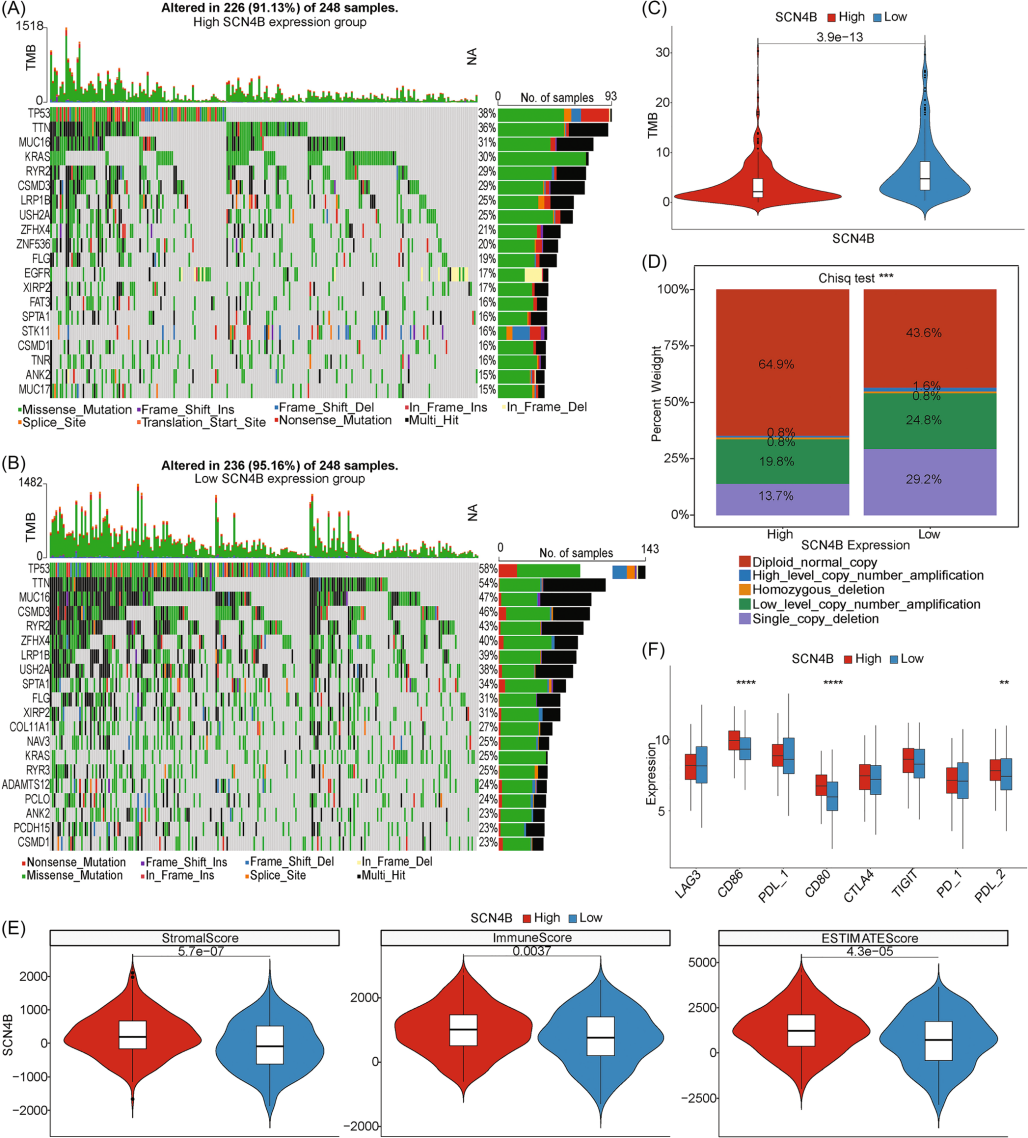
Gene mutation, immune checkpoints, and TMB in HEG and LEG. (A,B) TMB analysis in SCN4B HEG and LEG. (C) TMB difference between HEG and LEG. (D) CNV type differences between HEG and LEG. *** represents *p* ≤ 0.001. (E) Immune Score, ESTIMATE Score, and Stromal Score in HEG and LEG. (F) Expression of eight immune checkpoint genes in HEG and LEG.

Most human cancers are caused by somatic alterations, leading to oncogene activation or tumor suppressor gene inactivation. Systematic approaches based on sequences of the human genome have made identifying cancer genome alterations such as point mutations and copy number increases or decreases possible.^23^ The somatic mutation profile of TCGA‐LUAD was used to observe the difference in somatic mutation level between HEG and LEG, and the tumor mutation burden (TMB) was also calculated. Mutation results showed that the gene TP53 mutation rate was the highest in both groups (Figure 6A,B, high: 38%, low: 58%). TMB analysis showed that there was a significant difference between HEG and LEG (Figure 6C). Meanwhile, we analyzed the differences in CNV types between HEG and LEG and found that the proportion of Diploid_normal_copy in HEG (64.9%) was higher than that in LEG (43.6%). There were significant differences by chi‐square test in CNV types between HEG and LEG (Figure 6D).

### 3.7 Immunotherapy outcome prediction between different SCN4B expression groups

More and more patients with advanced lung cancer benefited from immunotherapy, which promoted us to screening immunotherapy candidates. Firstly, we estimated ESTIMATE Score, Immune Score, and Stromal Score by ESTIMATE and found these scores were significantly higher in the HEG group than LEG group (Figure 6E). Furthermore, we analyzed the difference of eight immune checkpoint genes PD‐1 (PDCD1), CTLA4, PDL‐1 (CD274), PDL‐2 (PDCD1LG2), CD80, CD86, LAG3, and TIGIT in HEG and LEG. Three immune checkpoint genes CD86, CD80, and PDL‐2 (PDCD1LG2) significantly upregulated in HEG compared to LEG (Figure 6F).

## 4 DISCUSSION

SCN4B was explored in this study, focusing on its function in LUAD. As a metastasis‐suppressor gene in many other cancers, SCN4B also inhibited the progression of LUAD. Higher expression of SCN4B indicated a better prognosis. Furthermore, we found two transcription factors, TAL1 and ERG, which might regulate SCN4B's expression by binding the upstream sequences.

We screened six out of 212 transcription factors that were highly expressed in LUAD samples by differential gene expression analysis, and three of them, TAL1, NFIX, and ERG, could regulate the expression of the SCN4B by binding to its upstream sequences. ChIP‐seq results in a further step verified the reliability of TAL1 and ERG regulating the expression of SCN4B by binding with it. TAL1, namely, T‐cell acute lymphocytic leukemia 1, also called SCL (stem cell leukemia), is a basic helix‐loop‐helix transcription factor (bHLHa17), which is essential in hematopoiesis.^24^ TAL1 is identified as one of the hub genes in the transcription network of LUAD, promoting the TGF‐β signaling pathway by upregulating the kinase insert domain receptor (KDR).^25^ TAL1 is frequently downregulated in LUAD because it may be silenced by hypermethylated CpG sites within its promoter region, supporting TAL1 as a potential tumor suppressor of LUAD.^26^ Another research also proves that in lung cancer patients, downregulation of TAL1 is negatively related to OS, suggesting TAL1's suppressing function in lung cancer,^27^ despite TAL1 is considered to be an oncogene in some diseases.^28^ Few studies are about the relationship between TAL1 and SCN4B, so our research provides a valuable finding that SCN4B may be one of the genes regulated by TAL1 to control the progression of LUAD. ERG encodes transcription factors of the erythroblast transformation‐specific (ETS) family, which play a central role in angiogenesis, inflammation, cell proliferation, differentiation and apoptosis, etc. It drives tumor progression and cancer‐related phenotypes.^29^ Although it is hard to be targeted for treatment as a transcription factor, its downregulation in LUAD together with SCN4B shows a little part of the mechanism involved LUAD progression. According to our GSEA results, 126 pathways were significantly enriched in HEG than in LEG. The top 10 activated pathways mostly focused on the main functions of TAL1 and ERG, such as hematopoietic cell lineage, complement and coagulation cascades, calcium signaling pathway, vascular smooth muscle contraction, and cell adhesion, indicating TAL1 and ERG might be hub nodes in the network of SCN4B and LUAD.

Genetic changes in cancer cells and rearrangement of tumor microenvironment components are key to cancer progression.^30^ In this study, the infiltration ratios of 13 types of immune cells in LUAD were significantly different between HEG and LEG. Among the 13 types, the score of activated memory CD4 T cells was negatively correlated with SCN4B expression, and the scores of RMCs and monocytes positively correlated with SCN4B expression. Scn5a/Scn4b VGSC is key to the positive selection of CD4+ T cells in the thymus by enabling the sustained entry of Ca^2+^ into CD4+CD8+ double‐positive thymocytes, and SCN4B does not express in mature single‐positive thymocytes or peripheral T cells.^31^ High mast cell abundance was correlated with prolonged survival in early‐stage LUAD patients and TP53 mutation,^32^ which is consistent with our results. Interestingly, RMCs are strongly associated with better OS, but activated mast cells are related to adverse survival. The RMC‐associated miRNAs work essentially in mRNA metabolic process, calcium modulating, p53 pathways, etc.^33^ A high infiltration ratio of immune cells with high SCN4B expression infers that SCN4B may aid in providing a relatively friendly tumor microenvironment for immunotherapy in LUAD. Our finding of higher expression levels of immune checkpoints CD86, CD80, and PDL‐2(PDCD1LG2) in HEG provided clues for the immunotherapy of LUAD.

## 5 CONCLUSIONS

In this study, we explored the influence of SCN4B on LUAD. SCN4B expresses higher in normal samples, and SCN4B is able to be an independent prognostic signature that higher expression predicts better prognosis for LUAD patients. Transcription factors TAL1 and ERG may regulate the expression of SCN4B by binding its upstream sequences. Our research provides deeper insight into how SCN4B influences the progression of LUAD and the prognosis and is valuable in improving the effectiveness of treatment in LUAD by suppressing metastasis.

## AUTHOR CONTRIBUTIONS

Minting Ma and Bin Guo contributed to the study conception and design. Minting Ma and Hongwei Lu guided the methods of the study. Minting Ma, Bin Guo, and Lei Hong performed the software. Minting Ma and Hongwei Lu analyzed the data. Bin Guo and Lei Hong collected the data. Minting Ma, Bin Guo, and Lei Hong drafted the original version of the article. Hongwei Lu and Lei Hong reviewed the article. Bin Guo and Hongwei Lu administrated the project. All authors read and approved the final manuscript.

## CONFLICT OF INTEREST STATEMENT

The authors declare that they have no competing interests.

## ETHICS STATEMENT

Not applicable.

## Supporting information


**Table S1.** The detailed results of GSEA.

**Table S2.** 212 transcription factors that were highly expressed in LUAD samples.

**Table S3.** Upstream binding sequences of SCN4B with TAL1, NFIX and ERG.

## Data Availability

The data that support the findings of this study are available in the Cancer Genome Atlas (TCGA; https://tcga-data.nci.nih.gov/tcga/) database, the maf file of LUAD and CNV data, Gene Expression Omnibus (GEO database; https://www.ncbi.nlm.nih.gov/geo/), the Human Protein Atlas (HPA; https://www.proteinatlas.org/) database, UCSC (http://genome.ucsc.edu/), and the JASPER database (https://jaspar.genereg.net/).
